# Being “penny-wise but pound foolish” in cancer immunotherapy research: the urgent need for mouse cancer models to reflect human modifying factors

**DOI:** 10.1186/s40425-016-0195-0

**Published:** 2016-12-20

**Authors:** William J. Murphy

**Affiliations:** Departments of Dermatology and Internal Medicine, UC Davis School of Medicine, Sacramento, CA USA

## Abstract

Inbred mice are the mainstay for preclinical cancer assessment of potential therapeutics, especially immune-based approaches. However, the use of young, lean, inbred mice housed under specific-pathogen-free conditions does not mirror the human cancer scenario. This commentary discusses some of the issues in evaluating immunotherapeutics in mice given recent advances.

## Main text

Immunotherapy is now at the forefront of cancer therapies with an increasing assortment of approaches being evaluated (ie checkpoint blockade, oncolytic viruses, chimeric antigen receptor (CAR) T and NK cells as well as dendritic cell vaccines and other immunostimulatory regimens alone or in combination). In addition, immunotherapy combined with radiation therapy and even chemotherapeutics have resulted in increased efficacy [1, 2] indicating that these combinations will also be increasingly applied. Given the increased potential of significant off-target toxicities arising (ie cytokine storm from the therapy, tumor lysis syndrome, autoimmune attack of normal cell-types) as well as the numerous factors that can impact efficacy, it is critical that preclinical models be used that can mirror the current human cancer scenario. Immunotherapy stands out as a regimen where a major variable is not only the cancer (as with radiation and chemotherapy application), but the patient’s own immune system and responsiveness, which is not always predictable, dynamic and is not easily assessed. Additionally, despite recent successes, many critical questions still remain: why do some patients respond and not others? Can the responses be sustained and amplified? What are potentially predictive surrogate markers for both responses and recurrence? What toxicities can be predicted and pre-emptively targeted? What is the best preclinical model to assess? Most of these questions revolve around the dynamics of immune system, which is constantly evolving in every individual. What makes immunotherapy also stand out (both in positive and negative ways) from other conventional cytoreductive therapies is the potential for continuous and sustained (if not amplified) responses after treatment.

Currently, the inbred mouse is by far the most commonly used preclinical model in cancer and it is growing in use, in part through the generation of genetically engineered mouse models (GEMM) that allow for spontaneous tumor generation rather than simple transfer of fully transformed and extensively cultured, mouse tumor lines into normal healthy young mice (although for treatment studies, transfer of the tumor lines are still predominantly used). It must be acknowledged immediately that critical discoveries, particularly in cancer immunotherapy, have been brought to clinical practice using this model. The basic characterizations of immune cells/pathways as well as fundamental principles in tumor progression and evasion (including multiple approaches in immune-targeted attack of the cancer) have all been borne out using these models. The issues arise when attempting to dig further and optimize these approaches and assess long-term impact. Importantly, the vast majority of preclinical mouse studies use relatively young (8–12 week old) healthy inbred mice; predominantly female (due to ease in housing) and housed under strict specific pathogen free (SPF) conditions. Therein resides the paradox of attempting to mirror the human clinical scenario using mice under these conditions. We have recently demonstrated that other variables such age and obesity can markedly impact outcome after administration of systemic immunotherapy in mice where young mice can tolerate regimens that are quickly lethal for aged or obese mice [3, 4]. Despite the fact that cancer is considered a disease of the aged (over the age 55 is median time for diagnosis in U.S.) and obesity in the U.S. is reaching epic proportions, it is surprisingly difficult to perform studies using aged and obese mice due to cost, time and accessibility. These mice are very expensive and time-consuming to generate in-house and, unless one has a grant from the National Institute on Aging (NIA), extremely difficult to obtain in sufficient numbers to perform efficacy studies. Obtaining these mice from commercial vendors can also be highly costly and unpredictable to impossible. Combine these issues with the inherent cost of performing long-term efficacy studies due to the need for larger sample size and the new NIH mandate to assess effects in both sexes, it is perhaps no wonder there is a paucity of literature assessing the impact of age and obesity on cancer progression and therapy outcome in mice despite the fact these factors predominate clinical cancer patients. There are even less studies assessing toxicities that can arise from the different therapies despite the fact these are growing both in incidence and severity in the clinic. Another key issue that further complicates mouse studies is the microbiome and impact of housing conditions on the immune response. Recent studies have clearly demonstrated the dramatic impact of the microbiome, not only immunotherapy but also on chemotherapeutic responses in mice [5–7]. These studies also demonstrate a critical issue in using inbred mice: microbiome variability between vendors and even institutional colonies can lead to false-positive or false-negative results in immunotherapy or even tumor growth using the same genetic strain. The microbiome variable may also account for differences in data and lack of reproducibility between laboratories being observed. Aging and obesity also impact the microbiome although these studies are even more preliminary. The NIH has implemented new guidelines for reagent authentication yet the emphasis has been on cell lines and reagents and not mice (other than assessment of both sexes unless justified). Genetic drift among mouse colonies is well-accepted but microbiome differences also influence differences in immune therapy outcomes using the same inbred strain, particularly when mice housed under SPF conditions are used. While SPF colonies have led to marked improvements in maintaining breeding colonies and reducing animal costs, it also has a tremendous impact on immune development, leading to an experimental model system which is even more remote immunologically from the human scenario. This has recently been dramatically brought to light immunologically comparing inbred mice housed under SPF conditions to conventionally-housed store-bought mice and even feral mice in which marked effects were observed [8]. Human exposure to pathogens throughout life all contribute to the immune fingerprint that is unique to the individual and that becomes even more fixed with normal aging and thymic involution. Additionally, latent infections that permeate the majority of the human population such CMV and EBV markedly impact T-cell function and result in significant skewing of the repertoire with aging. All of these effects result in an immune phenotype dramatically different from a young SPF inbred mouse. Xenograft studies using human immune cells in immunodeficient mice fare no better due to species-specific properties of key cytokines/factors (interferon-gamma and GM-CSF come to mind) and MHC divergence; which are critical for immune development, as well as potential for xenoreactivity and donor variability make such studies difficult to reproduce and clinically extrapolate. To better emulate the human clinical situation and the impact it will have on immunotherapy outcome, it will be necessary to perform studies in conventionally housed mice (it is of interest that once animal colonies became SPF in the 1980′s, graft-versus-host disease models became very difficult to repeat from earlier studies, an observation illustrating the impact of the environment on disease pathobiology). Ironically, it is currently exceedingly difficult to obtain and study either feral mice or even mice housed under conventional conditions due to common restrictions in place at the vast majority of SPF colonies. When attempting to assess effects of immunotherapeutic interventions, young lean mice kept under SPF conditions are far removed immunologically from the aged, obese cancer patient that has been exposed to a world full of pathogens over a lifespan. These epigenetic and environmental factors critically need to be incorporated into preclinical cancer models, especially when evaluating immune therapeutics. Using an inappropriate model can dramatically under or over-estimate efficacy as well as toxicities and mislead physicians when they try to apply the lessons learned in the laboratory.

If, as it seems, immunotherapy is going to become a mainstay of cancer treatment and be applied with other regimens or in combinations, it is absolutely imperative that the mouse modeling better reflect the dynamic human host-tumor relationship and immune phenotype. The genetic differences between mouse and man are a formidable barrier not easily circumvented but not including these other non-genetic variables (ie age, obesity, use of non-SPF housing conditions) only increases the magnitude of the barrier. NIH needs to provide funding resources encouraging more accessibility as well as optimization of such models as this crosses the different NIH institutes since immune/inflammatory pathways permeate almost every pathological condition. Such an approach will certainly increase the costs of doing research; however, clinically applicable research findings will spare patients enormous harm and costs from testing interventions that won’t help them and could possibly hurt them. Which cost is greater?

## Abbreviations

CMV: Cytomegalovirus
EBV: Epstein-Barr virus
GM-CSF: Granulocyte macrophage colony-stimulating factor
NIH: National Institute for Health
